# Phenotypes of streptozotocin-induced gestational diabetes mellitus in mice

**DOI:** 10.1371/journal.pone.0302041

**Published:** 2024-04-16

**Authors:** Narumi Takahashi, Osamu Ichii, Masaya Hiraishi, Takashi Namba, Yuki Otani, Teppei Nakamura, Yasuhiro Kon

**Affiliations:** 1 Laboratory of Anatomy, Department of Basic Veterinary Sciences, Faculty of Veterinary Medicine, Hokkaido University, Sapporo, Japan; 2 Laboratory of Agrobiomedical Science, Faculty of Agriculture, Hokkaido University, Sapporo, Japan; 3 One Health Research Center, Hokkaido University, Sapporo, Japan; 4 Laboratory of Laboratory Animal Science and Medicine, Department of Applied Veterinary Sciences, Faculty of Veterinary Medicine, Hokkaido University, Sapporo, Japan; Ramon Llull University: Universitat Ramon Llull, SPAIN

## Abstract

Gestational diabetes mellitus (GDM) in human patients disrupts glucose metabolism post-pregnancy, affecting fetal development. Although obesity and genetic factors increase GDM risk, a lack of suitable models impedes a comprehensive understanding of its pathology. To address this, we administered streptozotocin (STZ, 75 mg/kg) to C57BL/6N mice for two days before pregnancy, establishing a convenient GDM model. Pregnant mice exposed to STZ (STZ-pregnant) were compared with STZ-injected virgin mice (STZ-virgin), citrate buffer-injected virgin mice (CB-virgin), and pregnant mice injected with citrate buffer (CB-pregnant). STZ-pregnant non-obese mice exhibited elevated blood glucose levels on gestational day 15.5 and impaired glucose tolerance. They also showed fewer normal fetuses compared to CB-pregnant mice. Additionally, STZ-pregnant mice had the highest plasma C-peptide levels, with decreased pancreatic islets or increased alpha cells compared to CB-pregnant mice. Kidneys isolated from STZ-pregnant mice did not display histological alterations or changes in gene expression for the principal glucose transporters (GLUT2 and SGLT2) and renal injury-associated markers. Notably, STZ-pregnant mice displayed decreased gene expression of insulin-receiving molecules (ISNR and IGFR1), indicating heightened insulin resistance. Liver histology in STZ-pregnant mice remained unchanged except for a pregnancy-related increase in lipid droplets within hepatocytes. Furthermore, the duodenum of STZ-pregnant mice exhibited increased gene expression of ligand-degradable IGFR2 and decreased expression of GLUT5 and GLUT12 (fructose and glucose transporters, respectively) compared to STZ-virgin mice. Thus, STZ-pregnant mice displayed GDM-like symptoms, including fetal abnormalities, while organs adapted to impaired glucose metabolism by altering glucose transport and insulin reception without histopathological changes. STZ-pregnant mice offer a novel model for studying mild onset non-obese GDM and species-specific differences in GDM features between humans and animals.

## Introduction

Patients with diabetes mellitus (DM), encompassing a range of metabolic disorders, typically exhibit chronic hyperglycemia as a primary symptom [1]. The International Diabetes Federation estimates that the global population with DM will reach 700 million by 2045 [2]. Insulin reduces blood glucose levels (BGLs) by facilitating glucose uptake into systemic tissues through glucose transporters, including facilitative (GLUTs) and sodium-coupled glucose transporters (SGLTs), which are encoded by solute carrier family members. DM is triggered by disturbed insulin secretion and/or alteration of the cell response to insulin via the insulin receptor (INSR) or the insulin like growth factor 1 receptor (IGF1R). Type 1 diabetes is characterized by deficient insulin secretion, while type 2 diabetes results from severe insulin resistance due to receptor dysfunction or subsequent signaling abnormalities. Additionally, DM occurs in middle- to old-aged dogs and cats, with its pathology resembling type 1 diabetes in dogs and type 2 diabetes in cats [3, 4].

In mammals, systemic maternal tissues undergo morphological and functional adaptations to accommodate increased blood supply and nutrient metabolism for fetal development. Throughout pregnancy, maternal beta cells within pancreatic islets proliferate, leading to heightened insulin secretion and resistance in maternal tissues, thereby regulating normal blood glucose levels in both humans and mice [5, 6]. Additionally, pregnancy increases renal blood flow and the glomerular filtration rate (GFR) in both human and murine models [7, 8]. These maternal adjustments are orchestrated by various hormones, including prolactin, which promotes beta-cell expansion in mice [6], and progesterone and relaxin, which contribute to increased GFR in humans and rats [9]. Pregnant women exhibit varying levels of urinary glucose excretion, influenced by alterations in the renal threshold for glucose. This threshold refers to the glucose concentration in the blood at which glucose appears in the urine [10]. the renal threshold for glucose decreases during pregnancy, likely due to enhanced renal blood flow and the downregulation of glucose transporters such as GLUT or SGLT in the proximal tubule, the primary site of glucose reabsorption within the nephron [11].

Glucose metabolic abnormalities during pregnancy are typically categorized as follows: 1) pre-existing diabetes DM complicating pregnancies, 2) cases of evident DM manifesting during pregnancy, and 3) gestational diabetes mellitus (GDM) in humans. Globally, their incidence is on the rise, particularly GDM, which affects 13–25% of pregnancies [12]. These conditions can lead to adverse outcomes such as miscarriage, fetal malformation, or macrosomia [13]. Moreover, the number of fetuses also influences pregnancy outcomes in women with GDM, with twin pregnancies associated with an increased risk of macrosomia [14]. The diagnosis of GDM typically relies on spot blood glucose levels or hemoglobin A1C and blood glucose levels during a glucose tolerance test (GTT), along with assessment for any DM-related organ damage. In murine models, DM during pregnancy has been shown to impact renal pathology via mitogen-activated protein kinase (MAPK) signaling. This results in the production of proinflammatory cytokines such as interleukin 6 (IL-6) [15]. Furthermore, pregnant mice with DM exhibit altered lipid metabolism, characterized by elevated serum levels of low-density lipoprotein (LDL), total cholesterol, triglycerides, and increased lipid droplets in the liver [16].

Patients diagnosed with GDM typically exhibit normal blood glucose levels and lack overt DM symptoms before pregnancy. However, they demonstrate a predisposition to DM, characterized by abnormal GTT results, and only develop DM during pregnancy. These clinicopathological features help distinguish GDM from pre-existing DM [17]. The NOD and Akita mouse strains serve as models for type 1 diabetes, whereas the KK strain is recognized for type 2 diabetes [18, 19]. Furthermore, the administration of streptozotocin (STZ) and alloxan in animals induces insulin-dependent DM by targeting pancreatic beta-cell death [18]. Consequently, these models have primarily been utilized for studying pre-existing DM. However, despite efforts involving a high-fat diet, STZ injections, or their combination to induce obesity and/or hyperglycemia during pregnancy from a virgin state, these phenotypes do not precisely mimic the pathology of GDM [20]. Moreover, obesity and a family history of DM are established risk factors for GDM development [13], whereas many Asian patients with GDM lack obesity symptoms [21]. Hence, suitable GDM models have not yet been established, and the causes, mechanisms, and effects of GDM on maternal tissues remain incompletely understood in both humans and animals.

This study established a novel mouse model mimicking GDM using a convenient STZ-injection-based method. The observed phenotypes included glucose tolerance abnormalities evident in GTT or during pregnancy. Furthermore, we elucidated the pathological changes affecting reproductive ability and the histology of maternal tissues. These findings offer fresh perspectives on glucose metabolism and abnormalities during pregnancy, bridging insights from both human and animal studies.

## Materials & methods

### Animals

We obtained thirty-one female C57BL/6N mice aged 14 weeks from Japan SLC, Inc. (Hamamatsu, Japan). All animal experiments were authorized by the Institutional Animal Care and Use Committee of the Faculty of Veterinary Medicine, Hokkaido University (approval no. 21–0008, 23–0062). Moreover, they were conducted following the Guidelines for the Care and Use of Laboratory Animals of Hokkaido University, Faculty of Veterinary Medicine, which is endorsed by the Association for Assessment and Accreditation of Laboratory Animal Care International.

### GDM induction

**Fig 1:**
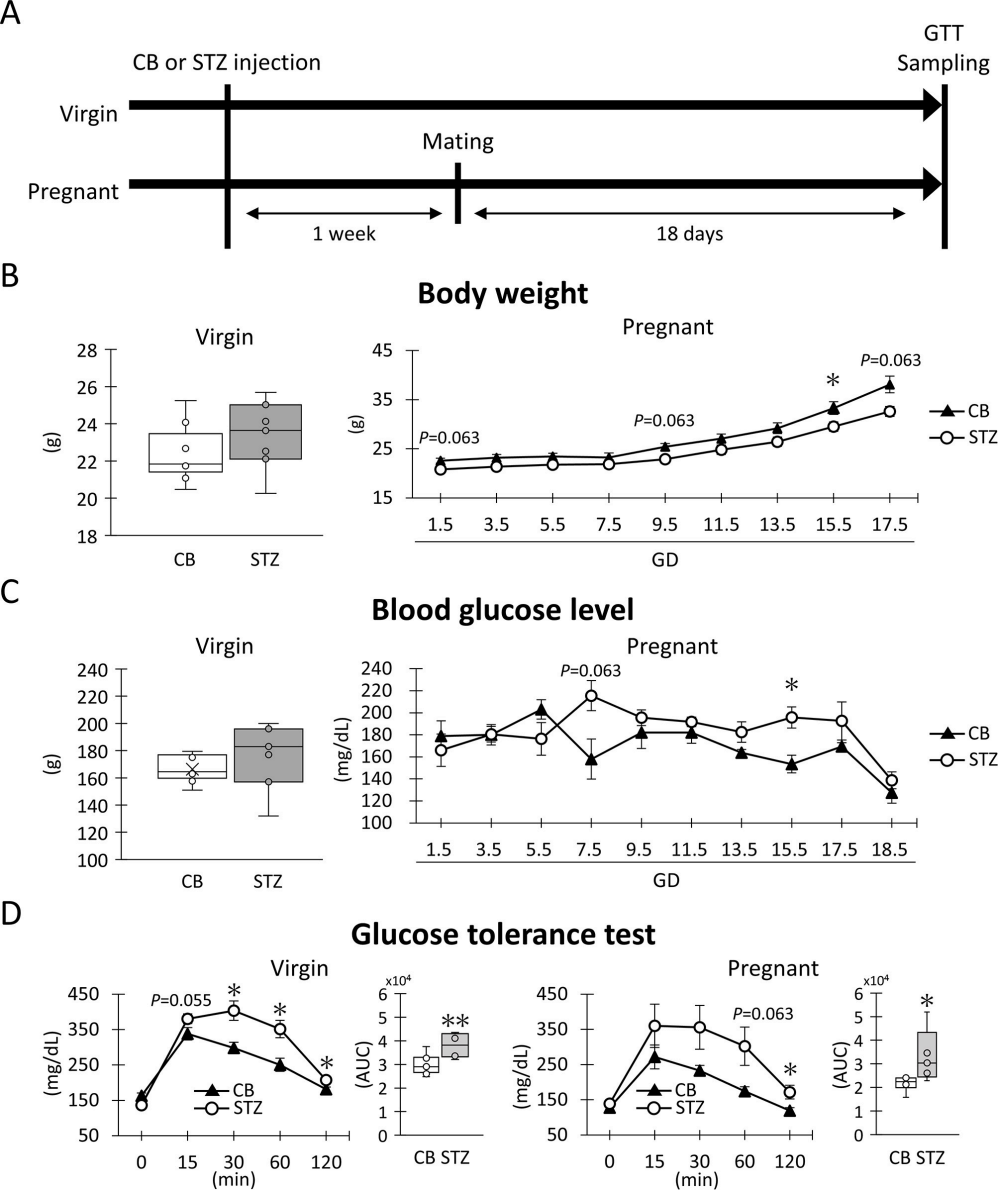
Experimental schedule and indices of non-obesity gestational diabetes mellitus. (A) Experimental schedule. (B) Body weight. (C) Blood glucose levels. These values were measured at one day before sampling day (virgin) and every other day during gestation (pregnancy). (D) Glucose tolerance test and area under the curve (AUC) for 0 to 120 min. These values were measured on sampling day. Box plot represents the minimum, first quartile, median, third quartile, and maximum (n > 4). Line graph represents the mean ± SE (n > 4). Significant differences: citrate buffer (CB) vs streptozotocin (STZ) in virgin or pregnant status, Mann-Whitney *U*-test (*: *P* < 0.05, **: *P* < 0.01).

STZ (75 mg/kg; FUJIFILM Wako Pure Chemical Corporation; Osaka, Japan, administered on two consecutive days) dissolved in 20 mM citrate buffer (CB; pH 4.7) or CB alone was intraperitoneally (i.p.) administered to virgin female mice at 14 weeks of age (CB-virgin, n = 9; STZ-virgin, n = 7; Fig 1A). Additionally, female mice similarly treated with CB or STZ were mated with males one week after i.p. administration: CB-pregnant (n = 9) and STZ-pregnant (n = 6). The day of observing the vaginal plug was designated as gestational day 0.5 (GD0.5).

Blood glucose levels (BGL) and body weight were measured during pregnancy every two days. BGL was assessed in tail vein blood using a Medisafe Fit (TERUMO Co., Ltd., Tokyo, Japan). At GD18.5, pregnant mice underwent a glucose tolerance test (GTT). Briefly, all mice were fasted for 6 hours from 8 am to 2 pm, followed by an intraperitoneal injection of a 0.2 g/mL glucose solution (D(+)-Glucose, FUJIFILM Wako Pure Chemical Co.) dissolved in phosphate-buffered saline (PBS) at a dose of 7.5 μL/g body weight. BGLs were measured before (0 min) and at 15, 30, 60, and 120 min after glucose injection. Blood samples collected at 0, 15, and 30 min post-injection were supplemented with 500 KIU/mL aprotinin (FUJIFILM Wako Pure Chemical Co.) and 10 U/mL heparin sodium (Mochida Pharmaceutical Co., Ltd.; Tokyo, Japan) to measure plasma C-peptide concentration.

### Sample collection

Pregnant mice treated with CB or STZ were euthanized at GD18.5 by cervical dislocation following the cutting of femoral arteries under deep anesthesia induced by an intraperitoneal (i.p.) injection of a mixture comprising 0.3 mg/kg medetomidine (Dorbene, Kyoritsu Seiyaku; Tokyo, Japan), 4 mg/kg midazolam (Dormicum, Astellas Pharma; Tokyo, Japan), and 5 mg/kg butorphanol (Vetorphale, Meiji Seika Pharma; Tokyo, Japan). Virgin mice of the same age as pregnant mice (18 weeks old) were euthanized using the same protocol. Following euthanasia, blood from the femoral artery and vein, urine, pancreas, liver, duodenum, kidney, ovary, and uterus, including the fetuses, were promptly collected for further analysis. The pancreas was divided into left and right lobes, with each lobe attached to the stomach and duodenum, respectively. Subsequently, the fetuses were separated from the uterus, and the number of fetuses and absorption scars were counted. Mean fetal weight values were then calculated using all fetuses from each mother. For RNA analysis, the liver, duodenum, and kidney samples were preserved in RNAlater (Thermo Fisher Scientific; Waltham, MA, USA).

### Blood and urine analysis

To evaluate the insulin secretion ability of each mother, plasma levels of C-peptide were quantified using a mouse C-peptide ELISA Kit (Cat.# M1304, Morinaga Institute of Biological Science, Inc.; Kanagawa, Japan). Urine was utilized to evaluate urinary glucose using multisticks pro 11 (Siemens Healthcare Diagnostics Co. Ltd.; Tokyo, Japan), where urinary glucose^+^ and urinary glucose^-^ were defined as scores 1 and 0, respectively. The mean of urinary glucose scores was calculated in each group.

### Histological analysis

The pancreas, liver, kidney, and ovary were fixed with 4% paraformaldehyde in 0.1 M phosphate buffer at 4°C overnight. Following fixation, the tissues were dehydrated using graded ethanol and embedded in paraffin. Sections with a thickness of 3 μm (for pancreas, liver, and ovary) or 2 μm (for kidney) were then cut. These sections of the pancreas, ovaries, liver, and kidneys were stained with hematoxylin-eosin (HE) or periodic acid-Schiff hematoxylin (PAS-H).

### Immunohistochemistry (IHC)

IHC was performed to detect glucagon (alpha cells) and proinsulin/insulin (beta cells) in the pancreatic islets, and Perilipin 2 (also known as adipose differentiation-related protein, ADRP) in the liver. The sections were deparaffinized, and antigen retrieval was conducted for pancreatic sections. Subsequently, to block internal peroxidase activity, the sections were immersed in methanol containing 0.3% H_2_O_2_ for 20 min at 25°C. After washing three times with PBS, the sections were incubated with blocking serum for 1 h at 25°C to prevent nonspecific reactions. Then, the sections were incubated with primary antibodies overnight at 4°C. Following incubation, the sections were washed thrice in PBS and incubated with biotinylated secondary antibodies for 30 min at 25°C. After three additional washes in PBS, the sections were incubated with streptavidin-conjugated horseradish peroxidase (SAB-PO kit, Nichirei; Tokyo, Japan) for 30 min at 25°C. Subsequently, the sections were washed three times in PBS, and the immune-positive reaction was visualized with 3,3′-diaminobenzidine tetrahydrochloride in 0.05 M Tris-HCl buffer-H2O2 solution. Finally, the sections were counterstained with hematoxylin. Details of antigen retrieval, dilution, and antibodies are provided in Table 1.

**Table 1:** IHC protocol used in this study.

Purpose	Antigen	Host	Dilution	Source	Retrieval	Blocking
Primary	Glucagon	Rabbit	1:500	ab18461, Abcam, Cambridge, UK	CB 115°C 15 min	10% NGS
	Insulin/proinsulin	Mouse	Undiluted	418091, Nichirei, Tokyo, Japan	NA	
	ADRP/Perilin2	Rabbit	1:200	15294-1-AP, Proteintech, Tokyo, Japan		
Secondary	Rabbit IgG (biotinylated)	Goat	Undiluted	SAB-RO(R) Kit, Nichirei, Tokyo, Japan		
	Mouse IgG (biotinylated)					

ADRP, adipose differentiation-related protein. NA, not applicable. CB, citrate buffer. NGS, normal goat serum.

### Histoplanimetry

HE-stained pancreatic sections (3 sections of each mouse, right and left lobes respectively), HE-stained ovarian serial sections (per 100-μm-thick of each mouse), and IHC-performed liver sections (1 section of each mouse) were converted to virtual slides using Nano Zoomer 2.0 RS (Hamamatsu Photonics Co., Ltd.; Hamamatsu, Japan). For HE-stained sections, NDP. view2 (Hamamatsu Photonics Co., Ltd.) was utilized to measure the area and number of pancreatic islets and corpora lutea. A BZ-X Analyzer (Keyence, Osaka, Japan) was employed to measure the pancreatic area. The measured values from each pancreatic lobe (over 30 islets randomly selected from the same HE-stained pancreatic 3 sections) were utilized for histoplanimetric analysis. Regarding IHC sections, glucagon^+^ or proinsulin/insulin^+^ areas were quantified using a BZ-X Analyzer (Keyence; over 35 islets of each lobe in a section from each mouse), and their area ratio to pancreatic islets was calculated. For liver analysis, ADRP/Perilipin 2^+^ lipid droplets having over 5 μm of diameter were counted (six fields of view; 300×300 μm^2^ in a section of each mouse).

### Quantitative polymerase chain reaction (qPCR)

Total RNA from the liver, duodenum, and kidney preserved in RNAlater solution (Thermo Fisher Scientific) was isolated using TRIzol reagent (Thermo Fisher Scientific). The extracted total RNA (500 ng/μL) was then utilized as a template for complementary DNA (cDNA) synthesis, employing ReverTra Ace qPCR RT Master Mix (Toyobo Co., Ltd; Osaka, Japan). Subsequently, qPCR analysis was conducted on the synthesized cDNA (20 ng/μL) utilizing Thunderbird SYBR qPCR Mix (Toyobo Co., Ltd) and the gene-specific primers detailed in Table 2. The primer pairs were meticulously designed and chosen based on stringent criteria: 1) ensuring that the primer pairs did not generate products smaller than 100–250 bp derived from non-specific genes, even if there were several complementarities between the target gene and others and 2) ensuring that more than five base sequences of the primers were distinct from sequences derived from non-specific genes. The qPCR cycling conditions comprised an initial denaturation step at 95°C for 1 minute, followed by 40 cycles of denaturation at 95°C for 15 seconds and annealing/extension at 60°C for 45 seconds. The obtained data were normalized to the values of beta-actin and CB-virgin using the delta-delta Ct method.

**Table 2:** List of primers used in this study.

Gene symbol	Accession	Primer sequence (5′-3′)		Product size
		Forward	Reverse	(bp)
*Actb*	NM_007393	TGTTACCAACTGGGACGACA	GGGGTGTTGAAGGTCTCAAA	165
*Igf1r*	NM_010513.2	TCGATTCGGTGACTTCTGC	AATGGCGGATCTTCACGTAG	156
*Igf2r*	NM_010515.2	ACTGTGACCGGACTACACAGA	CACTGTACCTTGACAGTGAGGAG	171
*Il1f6*	NM_019450	TCCTGCAGAACAATATCCTCAC	GTTCGTCTCAAGAGTGTCCAGA	104
*Il6*	NM_031168	CAACGATGATGCACTTGCAGA	GGTACTCCAGAAGACCAGAGGA	128
*Insr*	NM_001330056.1	AACGACATTGCCCTGAAGAC	TAGGGTTCCCACCTCAACAG	113
*Kim1/Havcr1*	NM_001166632	ACCAATGGACATCGTGTCACC	GGGTCTTCTTGGAGGACGTG	219
*Slc2a1*	NM_011400.3	AACATGGAACCACCGCTAC	GCCAACAGGTTCATCATCAG	171
*Slc2a2*	NM_031197.2	AGAGATCGCTCCAACCACAC	CAGCAGATAGGCCAAGTAGGA	149
*Slc2a3*	NM_011401.4	GCATTTGGCACACTAAACCA	TTGCAGGATAGCTGGAATGA	138
*Slc2a4*	NM_009204.2	CTTAGTAGAACGAGCTGGACGA	GACATAGCTCATGGCTGGAAC	124
*Slc2a5*	NM_019741.3	GAAGGAGGATGAGGCTGAGA	GAGGTAGATCTGATCGGCGTAG	163
*Slc2a6*	NM_172659.2	GCCTTGGTCTACACATCTCCA	CCAGGAGGTCATTGAGTAACA	157
*Slc2a7*	NM_001368869.1	AGCGACACGGAACATTCA	CGGCAGAGGTTATAGCGAAG	174
*Slc2a8*	NM_019488.4	CCTCACTCAACACCAGTACCAG	AGCTGCTGGAAGACCATGAG	183
*Slc2a9*	NM_001102414.1	ACAACCTCTCCGTGGTGAAC	TGGACACAGTCACAGACCAGA	129
*Slc2a10*	NM_130451.3	CCATCCATCCAGTCATCACA	ATCTCACTGAGGACCAACCAG	181
*Slc2a12*	NM_178934.4	TCTTTCCCGGTGGAATTAGA	AGCATACCCATGACAAACCA	126
*Slc5a1*	NM_019810.4	CCAGTAACATTGGAAGTGGTCA	AGATACTCCGGCATCGTCA	169
*Slc5a2*	NM_133254.3	GAGCAGAAGGTCCTGATTGA	ATGTTGCTGGCGAACAGAG	185
*Synpo*	NM_177340.2	CAAGCCTAGCTCTCTGGACC	GACACGGGGGAGATATCACTG	174
*Tgfb1*	NM_011577.2	ATGCTAAAGAGGTCACCCGC	TGCTTCCCGAATGTCTGACG	119
*Vegfa*	NM_001110266.1	GAGAGATGAGCTTCCTACAGCAC	TTCTCCGCTCTGAACAAGG	171

### Statistical analysis

The results are presented as box plots, illustrating the minimum, first quartile, median, third quartile, and maximum values, or as mean ± standard error in line graphs. Statistical analysis was conducted in a non-parametric manner. The significance between two groups was assessed using the Mann-Whitney *U*-test (*P* < 0.05). A chi-square test was employed to determine if there was a significant difference in urinary glucose levels between the two groups (*P* < 0.05). Additionally, the correlation between two parameters was examined using Spearman’s correlation coefficient (*P* < 0.05). Heat maps were generated using Microsoft Excel 2016 MSO software (Microsoft, Washington, USA).

## Results

### GDM induction in mice

The experimental protocol is summarized in Fig 1A. For GDM indices, including body weight, BGL, and GTT, were evaluated in the virgin and pregnant groups receiving CB or STZ (Fig 1B–1D). Regarding body weight, no significant difference was observed between the CB and STZ groups in virgin status, but CB-pregnant tended to show higher values at GD1.5, 9.5, 15.5, and 17.5 compared with STZ-pregnant, with significance detected at GD15.5 (Fig 1B). For BGL, no significant difference was observed between the CB and STZ groups in virgin status. However, STZ-pregnant tended to show higher values at GD7.5, and 15.5 compared with CB-pregnant, with significance detected at later time points (Fig 1C). GTT was conducted to evaluate glucose uptake ability in response to intraperitoneal glucose. In all examined groups, BGL increased 15 min after intraperitoneal glucose administration and then gradually decreased (Fig 1D). STZ-virgin tended to show higher values at 15 min and manifested significantly higher values at 30, 60, and 120 min after intraperitoneal glucose administration than CB-virgin. The area under the curve (AUC) for 0 to 120 min was significantly higher in STZ-virgin compared with CB-virgin. Furthermore, STZ-pregnant also tended to show higher values at 60 and 120 min after intraperitoneal glucose administration than CB-pregnant, with significance detected later. The AUC for 0 to 120 min was significantly higher in STZ-pregnant compared with CB-pregnant. Thus, STZ i.p. before pregnancy (75 mg/kg body weight, 2 consecutive days) did not alter BGL during virgin status but made GTT abnormalities apparent after glucose i.p. or during pregnancy.

### Reproductive ability in mice

**Fig 2:**
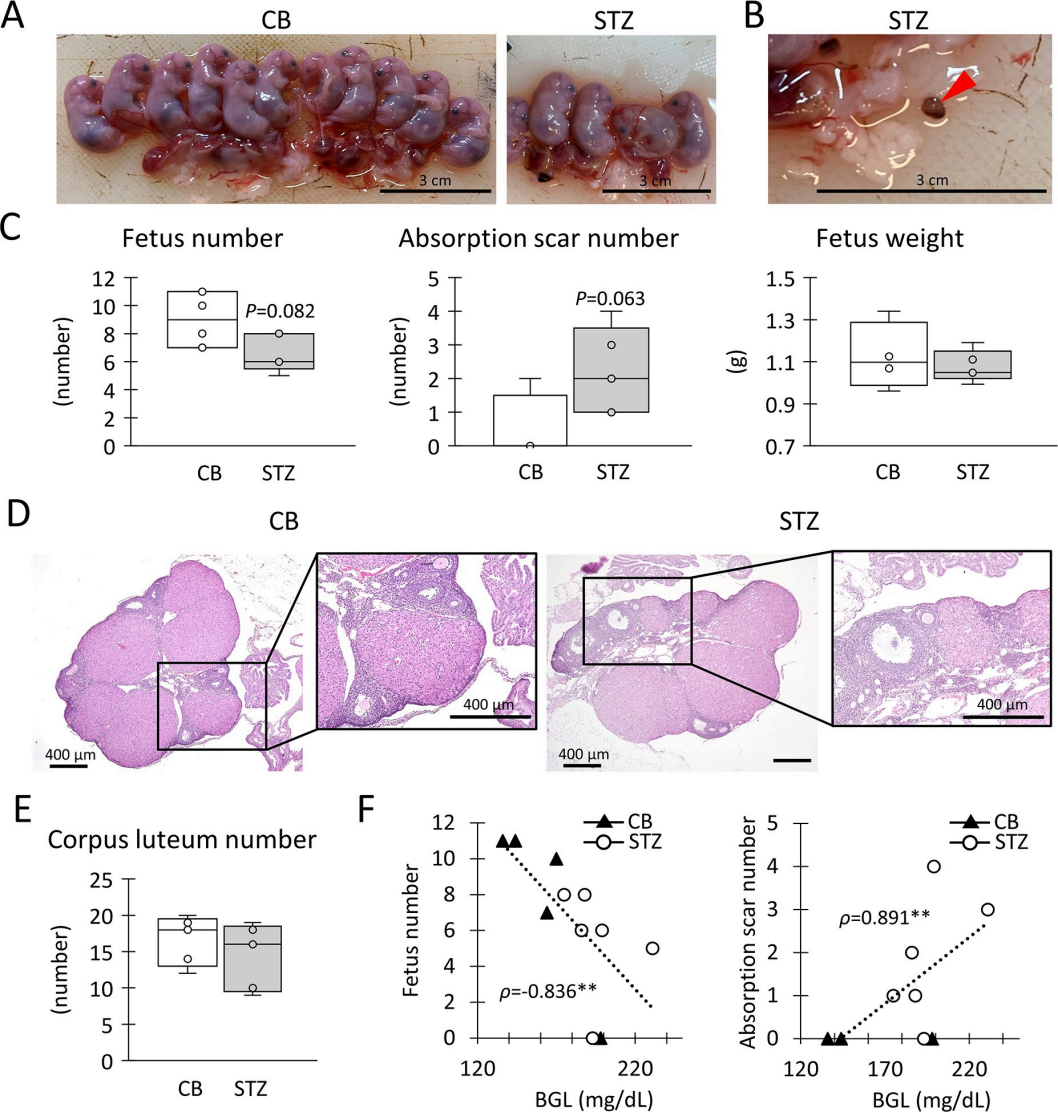
Indices of reproduction abilities. (A) Placentas and fetuses in citrate buffer (CB) or streptozotocin (STZ) injected pregnant. (B) Red arrowhead shows absorption scar in STZ-pregnant. Bars = 3 cm. (C) Each number of fetuses and absorption scars. Each weight of the fetus. (D) Histology of corpora lutea in ovary. Squares show the high magnification image of corpora lutea. HE staining. Bars = 400 μm. (E) Each number of corpora lutea. (F) Correlation between blood glucose level (BGL) vs fetus number or absorption scar number at 15.5 gestational days. Resolution of anatomical or histological images: 300 x 300 dpi. Box plot represents the minimum, first quartile, median, third quartile, and maximum (n > 4). Statistical analysis: CB vs STZ in virgin or pregnant status, Mann-Whitney *U*-test (*: *P* < 0.05). Significant correlation: Spearman’s correlation coefficient (**: *P* < 0.01).

To evaluate reproductive ability, the morphology of the fetuses, maternal uterus, and ovaries were compared between the CB-pregnant and STZ-pregnant groups. Fetal morphology was normal, with no malformations or stillbirths observed (Fig 2A); however, absorption scars were present in the uteri of both groups (Fig 2B). Particularly, CB-pregnant showed higher and lower numbers of fetuses and absorption scars, respectively, than STZ-pregnant (Fig 2C). However, fetal weight was comparable between the CB and STZ groups. No histological differences in the ovaries were observed between the groups (Fig 2D), and the number of corpora lutea, an index of the number of ovulations, was comparable between the groups (Fig 2E). Thus, STZ-pregnant exhibited decreased fetal numbers and increased absorption without any changes in fetal and ovarian morphology and ovulation. S1 Table summarizes the correlation between BGL and indices for reproductive abilities, with representative results shown in Fig 2F. Notably, BGL negatively and positively correlated with the fetus number and absorption scar number at GD15.5, respectively.

### Endocrine ability of pancreatic islets in mice

**Fig 3:**
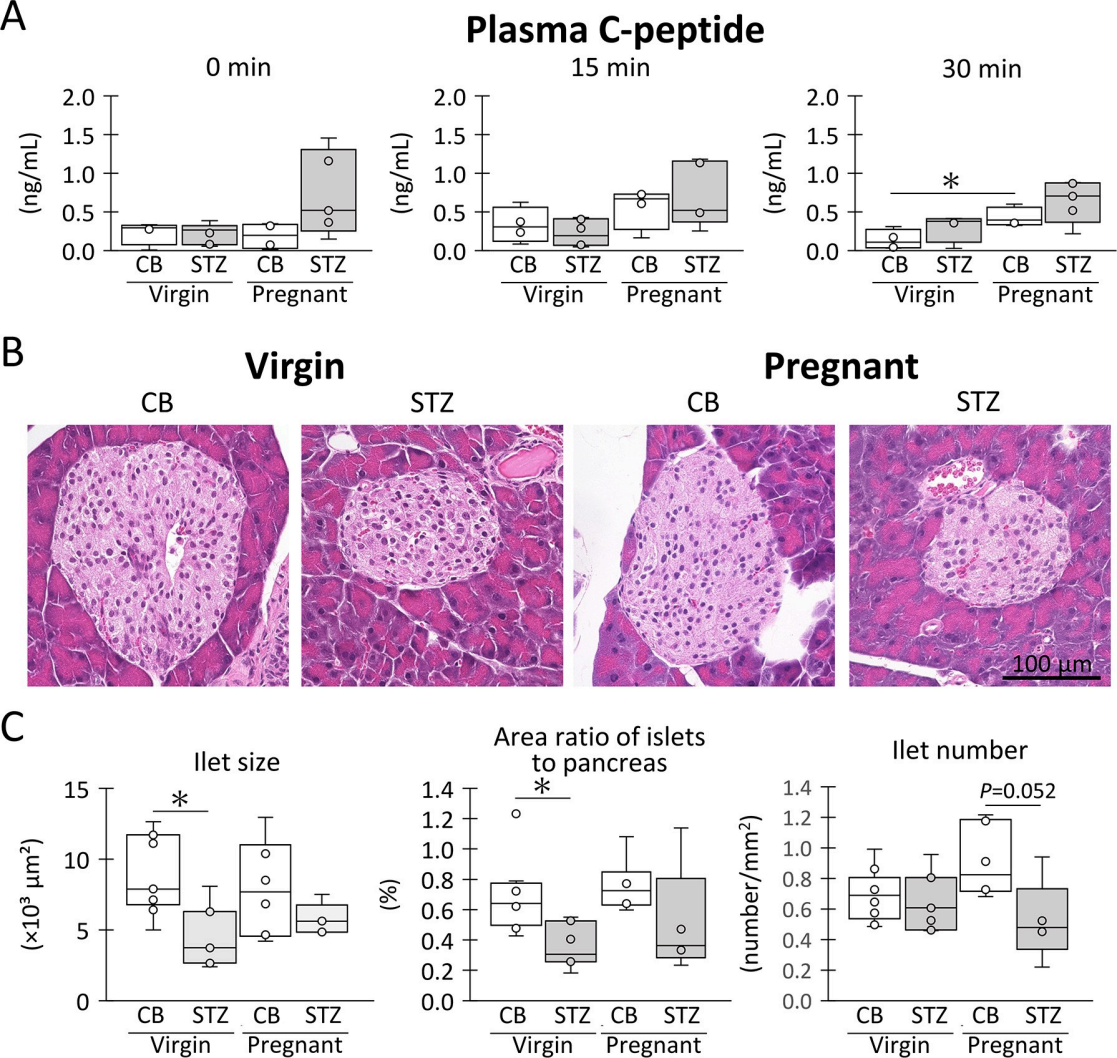
Evaluation of insulin secretion ability. (A) Plasma C-peptide level. (B) Histological feature of pancreatic islets. HE staining. Bars = 100 μm. Resolution of histological images: 300 x 300 dpi. (C) Pancreatic islet size, area ratio of islets to pancreas, and the number of pancreas islets. Box plot represents the minimum, first quartile, median, third quartile, and maximum (n > 4). Statistical analysis: citrate buffer (CB) vs streptozotocin (STZ) in virgin or pregnant status, and virgin vs pregnant in CB or STZ groups, Mann-Whitney *U*-test (*: *P* < 0.05).

As shown in Fig 1D, the GTT results suggested altered endocrine ability of the pancreatic islets in the STZ-pregnant mice. Therefore, Fig 3A shows the plasma C-peptide concentration as an index of insulin secretion during the GTT. CB-pregnant showed higher values 15 min after intraperitoneal glucose administration than CB-virgin, with significance observed at 30 min. Similarly, STZ-pregnant showed the highest values among the groups at 30 min, although there were no significant differences compared with STZ-virgin (*P* < 0.05 vs CB-virgin at 30 min).

As shown in Fig 3B, the pancreases of all examined groups did not exhibit clear histopathological changes, such as cell infiltration or cell death, around or inside the islets, except for decreased islet size in the STZ groups. For histoplanimetry (Fig 3C), the STZ groups tended to have decreased pancreatic islet sizes and area ratios of pancreatic islets compared to the CB groups, regardless of pregnancy, with significant differences detected among the virgin groups. No significant differences were observed between the CB-pregnant and STZ-pregnant groups in either parameter. The number of islets tended to increase during pregnancy in the CB group but not in the STZ group.

**Fig 4:**
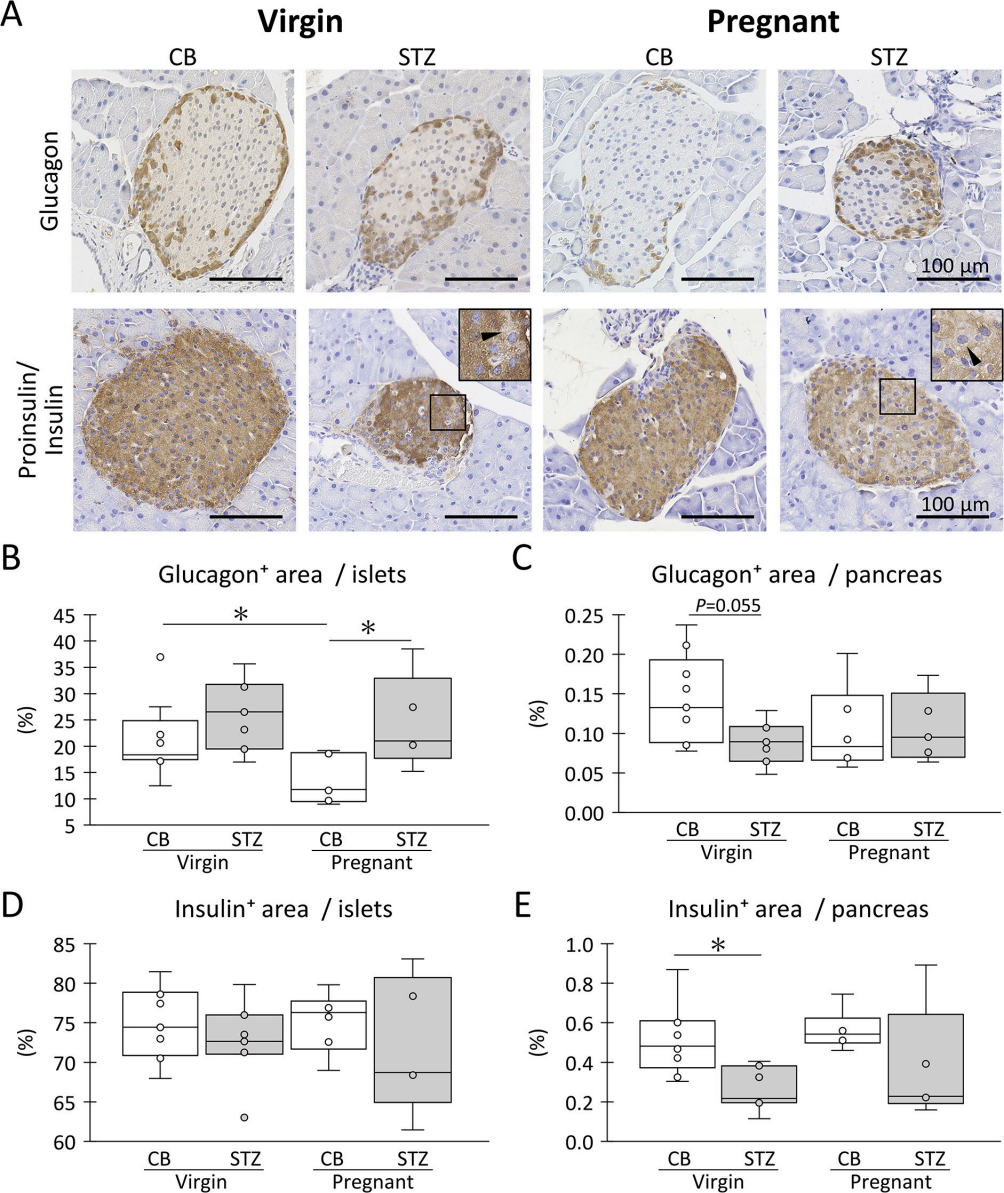
Morphology of pancreatic islets. (A) Histological features of glucagon^+^ cells (alpha cells) and insulin/proinsulin^+^ (beta cells) in the pancreatic islets. Inset magnifies insulin/proinsulin^-^ cells in squared area (arrowhead). Bars = 100 μm. Resolution of histological images: 300 x 300 dpi. (B) Ratio of glucagon^+^ areas to pancreatic islets. (C) The ratio of glucagon^+^ areas in the pancreas. (D) Ratio of insulin/proinsulin^+^ areas to pancreatic islets. (E) The ratio of the insulin/proinsulin^+^ area to the pancreas. Box plot represents the minimum, first quartile, median, third quartile, and maximum (n > 4). Statistical analysis: citrate buffer (CB) vs streptozotocin (STZ) in virgin or pregnant status, and virgin vs pregnant in CB or STZ groups, Mann-Whitney *U*-test (*: *P* < 0.05).

For further evaluation, IHC was performed for glucagon and proinsulin/insulin (Fig 4A). In all groups, glucagon^+^ alpha-cells were localized peripherally to the islets and seemed to be less abundant in CB-pregnant than in the other groups. Compared to glucagon^+^ alpha-cells, proinsulin/insulin^+^ beta cells are abundantly localized in the entire islet area. Notably, several cells weakly positive for proinsulin and insulin were detected in the STZ group. For the area ratio of glucagon^+^ alpha cells in the islets, CB-pregnant showed the lowest values, with significantly high values detected for CB-virgin and STZ-pregnant (Fig 4B). Furthermore, CB-virgin showed the highest values for the area ratio of glucagon^+^ alpha cells in the pancreas, with a higher tendency compared to STZ-virgin, and the values for other groups were comparable (Fig 4C). For the area ratio of proinsulin/insulin^+^ beta-cells in islets, no significant changes were observed regardless of STZ i.p. or pregnancy (Fig 4D). In contrast, the area ratio of proinsulin/insulin^+^ beta cells in the pancreas tended to decrease with STZ i.p., regardless of pregnancy, and a significant decrease was detected in the virgin group (Fig 4E).

### Gene expressions associated with glucose tolerance in mice

**Fig 5:**
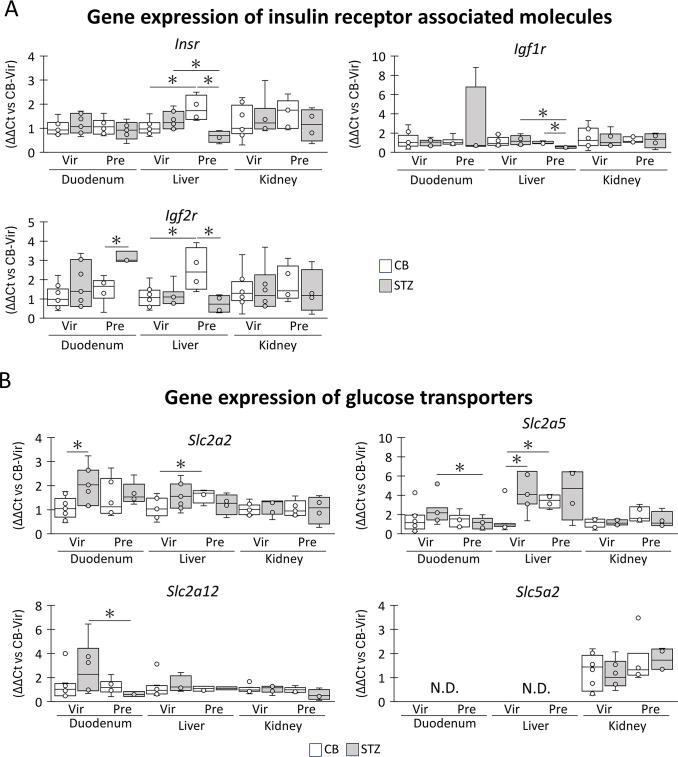
Genes associated with insulin sensitivity and glucose transportation. (A) mRNA expression of genes of insulin receptors. (B) mRNA expression of genes of glucose transporters. Representative results are extracted from the S1 Fig. Box plot represents the minimum, first quartile, median, third quartile, and maximum (n > 4). Statistical analysis: citrate buffer (CB) vs streptozotocin (STZ) in virgin (Vir) or pregnant (Pre) status, and Vir vs Pre in CB or STZ groups, Mann-Whitney *U*-test (*: *P* < 0.05). CB-Vir: CB-injected virgin. N.D.: not detected.

The expression of genes involved in glucose tolerance was analyzed in the duodenum, liver, and kidneys using qPCR (Fig 5). *Insr*, *Igf1r*, and *Igf2r* were examined to assess insulin or insulin-like function [22] (Fig 5A). In the CB group, *Isnr* expression tended to increase during pregnancy in the liver and kidneys, but not in the duodenum, with significant increase being detected in the liver. However, the *Isnr* expression of STZ-pregnant tended to be lower than that of the others in all examined organs, and a significant difference with STZ-virgin or CB-pregnant was observed in the liver. *Igf1r* expression tended to increase in the duodenum of STZ-pregnant mice, but in the liver, it was significantly decreased in STZ-pregnant compared to that in CB-pregnant or STZ-virgin, while that of others was comparable among the groups. *Igf2r* expression in the liver and kidneys of the CB group tended to increase during pregnancy, and significance was detected in the liver. STZ-pregnant showed significantly higher expression of *Igf2r* in the duodenum than STZ-virgin, but a lower trend was observed in the liver and kidney. Furthermore, a significant difference was detected in the liver compared with STZ-virgin.

To evaluate glucose incorporation from the blood, the expression levels of glucose transporter genes (13 members of the solute carrier family members) were compared among the groups (S1 Fig). Fig 5B summarizes the genes that showed significant expression changes by pregnancy and includes *Slc5a2*, which codes for SGLT2, a crucial molecule for glucose reabsorption in the kidney. Specifically, *Slc2a2* expression was significantly increased by STZ injection in the duodenum of the virgin group and by pregnancy in the liver of the CB group. *Slc2a5* expression was significantly decreased by pregnancy in the duodenum of the STZ group but increased by pregnancy in the liver of the CB group or by STZ injection in the virgin group. *Slc2a12* expression was significantly decreased by pregnancy in the duodenum of the STZ group. No significant changes were observed in all examined kidney genes (S1 Fig, Fig 5B).

### Changes due to GDM in maternal organs

**Fig 6:**
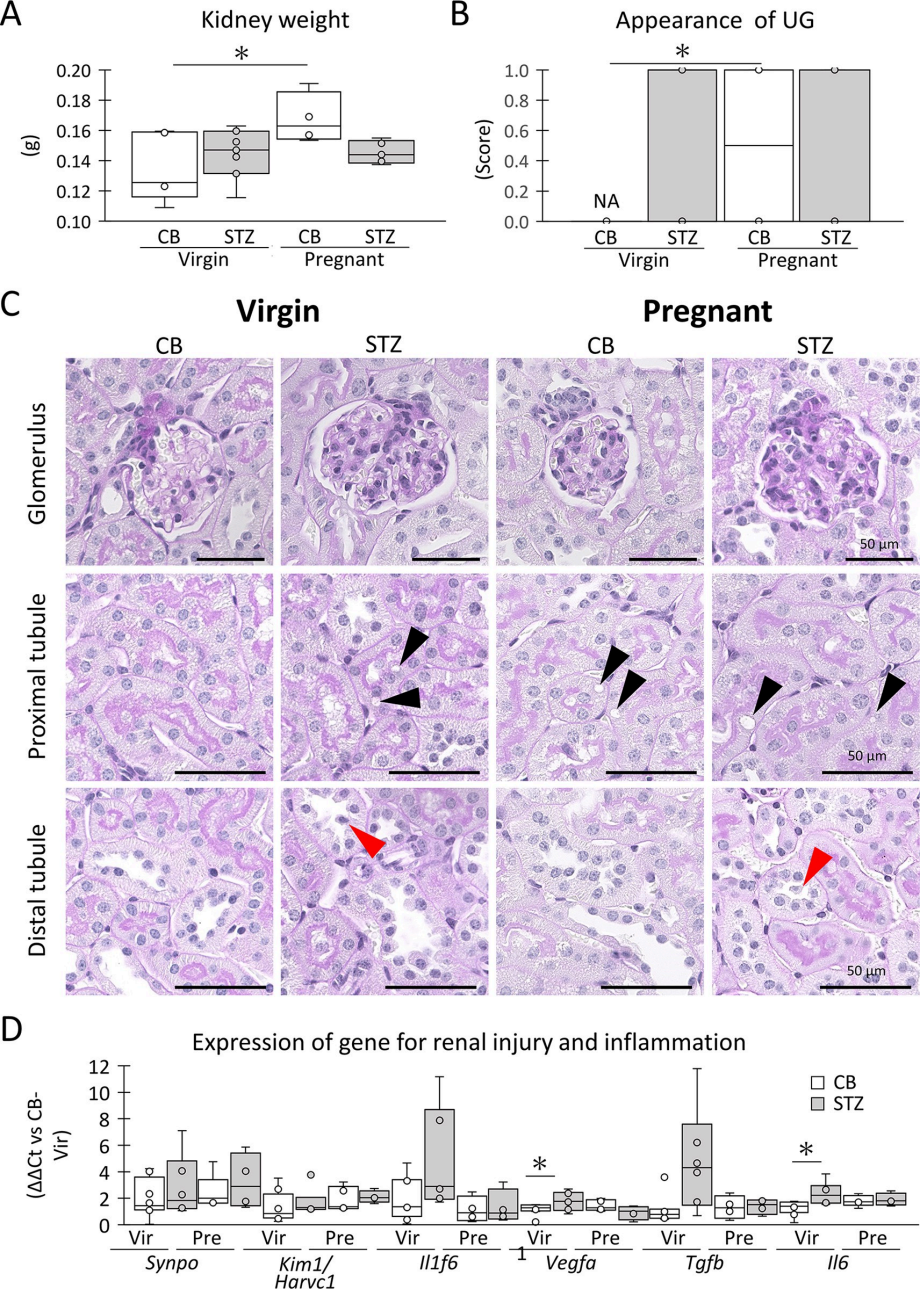
Maternal kidney changes due to STZ administration and pregnancy. (A) The weight of kidney. (B) Appearance score of mice showing urinary glucose. The mice indicating the presence of urinary glucose are scored as 1, while those not indicating are scored as 0. (C) Histology of kidney. Black arrowheads show vacuolar structure in epithelial cells. Red arrowheads show epithelial cell shedding. Bars = 50 μm. Resolution of histological images: 300 x 300 dpi. (D) mRNA expression of genes associated with inflammation and injury. Box plot represents the minimum, first quartile, median, third quartile, and maximum (n > 4). Statistical analysis: citrate buffer (CB) vs streptozotocin (STZ) in virgin (Vir) or pregnant (Pre) status, and Vir vs Pre in CB or STZ groups, Mann-Whitney *U*-test (*: *P* < 0.05). CB-Vir: CB-injected virgin. A chi-square test are performed to analyze whether there was a significant difference in the urinary glucose between the two groups (*P* < 0.05).

The maternal liver and kidney were analyzed as target organs for GDM [15, 23]. Kidney weight significantly increased due to pregnancy in the CB group, but not in the STZ group, with no significant difference observed between the CB and STZ groups in virgin and pregnant mice (Fig 6A). Urinary glucose appeared in the STZ-virgin, CB-pregnant, and STZ-pregnant groups without clear group differences; however, it was not detected in the CB-virgin group (Fig 6B). In the glomeruli, hypercellularity, membranous lesions, and hypertrophy were not observed in any of the groups (Fig 6C). In proximal tubules, vacuolar structures appeared in several tubular epithelial cells due to STZ i.p. or pregnancy. In the distal tubules, a few epithelial cells exhibited deciduous features in the STZ i.p. group (Fig 6C). Additionally, renal gene expression for cell injury and inflammation was analyzed by qPCR. STZ-virgin showed significantly higher expression of *Il6* and *Vegfa* than CB-virgin, but no changes due to pregnancy were detected in either group (Fig 6D).

**Fig 7:**
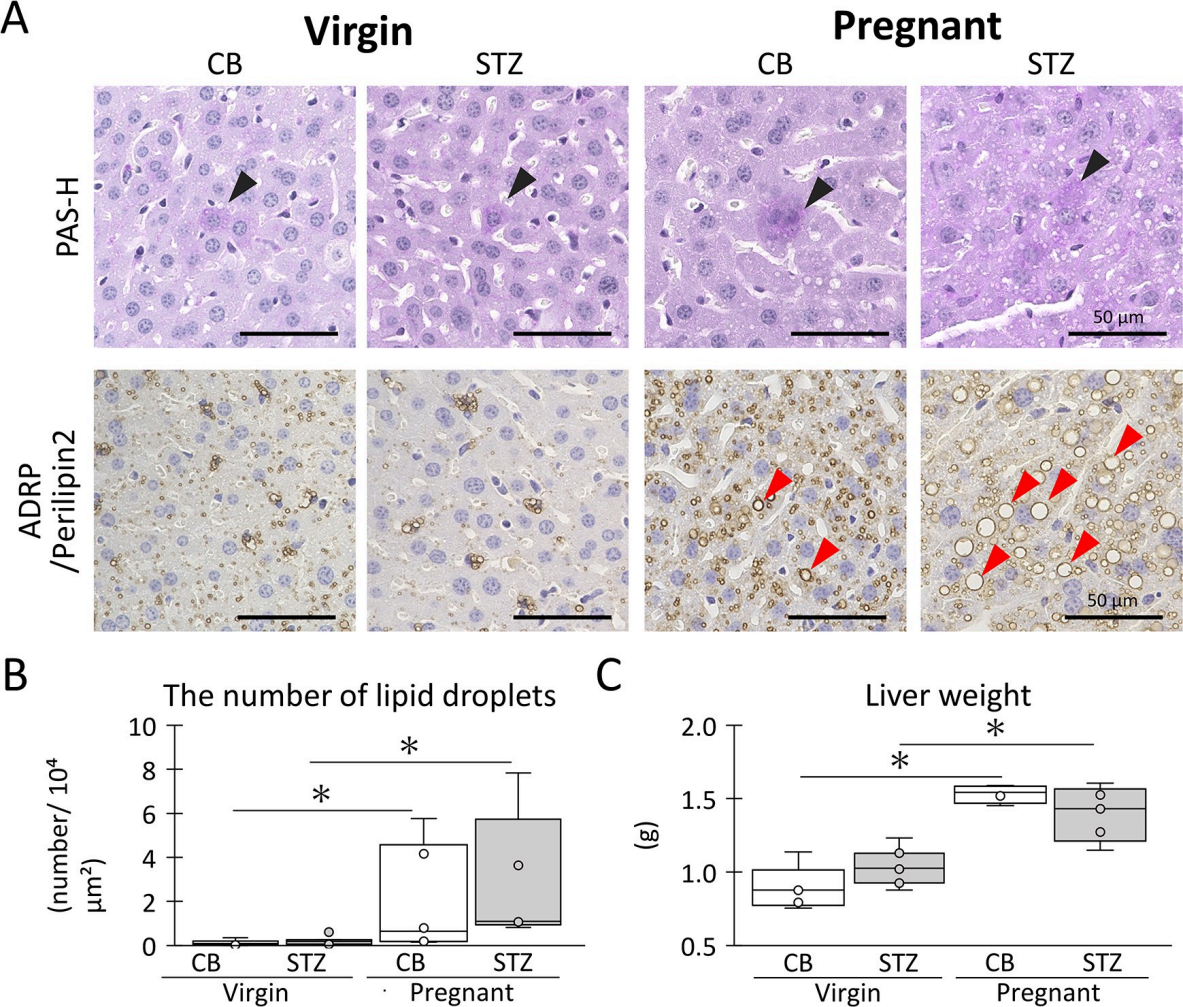
Maternal liver changes due to STZ administration and pregnancy. (A) Histology of liver. Black arrowheads show PAS^+^ cells. Red arrowheads show ADRP/ Perilipin2^+^ cells. (B) Number of large lipid droplets. (C) Liver weight. Each bar represents the mean ± SE (n > 4). Bars = 100 μm. Resolution of histological images: 300 x 300 dpi. Box plot represents the minimum, first quartile, median, third quartile, and maximum (n > 4). Statistical analysis: citrate buffer (CB) vs streptozotocin (STZ) in virgin or pregnant status, and virgin vs pregnant in CB or STZ groups, Mann-Whitney *U*-test (*: *P* < 0.05).

As shown in Fig 7A, cell infiltration, cell death, and accumulation of PAS^+^ materials were not evident in the livers of any of the examined groups. In contrast, ADRP/perilipin 2^+^ lipid droplets showed a clear increase during pregnancy in both CB and STZ groups. Additionally, STZ-pregnant tended to show increased sizes of ADRP/perilipin 2^+^ adipose lipid droplets compared with CB-pregnant (Fig 7A). The number of large ADRP/perilipin 2^+^ lipid droplets increased significantly during pregnancy in both the CB and STZ groups (Fig 7B). Pregnancy significantly increased liver weight compared with the virgin status regardless of STZ i.p., and no significant difference was observed between CB and STZ in both groups (Fig 7C).

## Discussion

In this study, mice exhibited a predisposition to GDM, as evidenced by impaired glucose tolerance following STZ injection in the virgin state. However, during pregnancy, they displayed hyperglycemia and reduced fertility due to impaired glucose tolerance. Analysis of this mouse model suggested that impaired glucose tolerance resulted from insufficient insulin secretion, attributed to the lack of an increase in islet number associated with pregnancy and reduced insulin sensitivity in maternal tissues. Additionally, altered lipid metabolism during pregnancy, as indicated by fat accumulation in the liver, may contribute to the development of GDM. These symptoms closely resembled those observed in non-obese women with GDM [24]. Renal lesions were indistinct, suggesting either a reserved capacity for cell injury by GDM or potential ameliorative effects conferred by pregnancy.

In humans, the diagnosis of DM is based on hyperglycemia or elevated blood glucose levels during the GTT. GDM is defined as impaired glucose tolerance during pregnancy that does not meet the criteria for DM. Typically, patients with GDM exhibit abnormal GTT scores at 0, 60, and 120 min (e.g., > 153 mg/dL at 120 min), although the threshold for hyperglycemia is lower than that of non-pregnant individuals with DM (e.g., > 200 mg/dL at 120 min) [25]. STZ is commonly used to induce animal models of DM due to its cytotoxic effects on pancreatic beta cells. However, it has been challenging to replicate the subtle pathology of human GDM. In our preliminary study, a single i.p. dose of STZ at 50 or 100 mg/kg body weight did not induce hyperglycemia or GTT abnormalities during pregnancy. However, a dose of 200 mg/kg body weight i.p. led to abnormalities even in the virgin state, indicating the failure to establish a GDM model. Previous studies have utilized low doses of STZ i.p. (55–75 mg/kg body weight) administered over five consecutive days to induce DM [26]. Therefore, we modified this approach to administer STZ i.p. before pregnancy (75 mg/kg body weight, two consecutive days). This low dose and relatively infrequent administration of STZ i.p. induced GTT abnormalities only after glucose i.p. administration or during pregnancy. Other studies have attempted to induce GDM in mice by feeding them a high-fat diet in conjunction with STZ i.p.; however, these mice often exhibited obesity. Given the increasing prevalence of non-obese patients, particularly among Asian pregnant women [21], the convenient STZ i.p. method developed in our study could be valuable for inducing non-obese GDM phenotypes in mice and for conducting detailed pathological analyses.

Abnormal glucose metabolism in GDM is primarily attributed to severe insulin resistance in maternal tissues, resulting from relative insulin insufficiency [17]. In humans, factors such as decreased secretion of adiponectin from adipose tissue in obesity [27] and genetic predispositions, including race or family history, contribute to insulin resistance [21]. However, the GDM mice utilized in our study exhibited a normal genetic background and body weight. Therefore, the GDM-like symptoms observed in these mice were primarily driven by enhanced insulin resistance in their tissues. This was evidenced by abnormalities observed in the GTT, decreased expression of insulin-related molecules in the examined organs, and consistently elevated blood C-peptide levels during GTT.

STZ selectively damages beta cells via GLUT2 by inducing DNA fragmentation [28], as evidenced by the decrease observed in STZ-virgin mice. Furthermore, the STZ-treated group exhibited several cells weakly positive for proinsulin/insulin, suggesting a reduction in insulin storage within beta cells. However, the CB group did not display changes in islet size or beta-cell composition, although there was a decrease in glucagon^+^ cells within islets during pregnancy. These findings indicate an increased number of islets containing beta cells in pregnant mice. Indeed, pregnancy is known to stimulate an increase in the number and function of beta cells to adapt to insulin resistance, leading to elevated insulin secretion [6, 29]. However, the STZ-pregnant group exhibited significantly lower numbers of islets containing beta cells and higher areas of glucagon^+^ cells compared to the CB-pregnant group. This suggests that the increase in islets containing beta cells typically induced by pregnancy did not occur in the STZ-pregnant group.

Notably, an imbalance between glucagon and insulin production has been implicated in patients with diabetes mellitus [30]. Moreover, recent studies have identified various progenitor beta cells in the pancreas, including an intermediate type between beta cells and alpha cells, as well as beta cells derived from alpha cells or pancreatic duct cells [31]. Therefore, it is plausible that pregnant mice differentiate these immature beta cells into mature beta cells to adapt to enhanced insulin secretion, and these differentiated cells may not be entirely detected. Importantly, i.p. administration of STZ (75 mg/kg, twice) might have impaired this differentiation process. These alterations in islets or beta cells in the pancreas contribute to the enhanced glucose resistance observed in STZ-treated pregnant mice.

Hyperglycemia is associated with infertility in humans [32] and with adverse preimplantation or embryogenesis in mice [33]. Human GDM causes miscarriage, malformations, or macrosomia of the fetus depending on the onset of hyperglycemia [13]. The estimated ovulation and fetal weight in STZ-pregnant were comparable to those in CB-pregnant, but STZ-pregnant exhibited a decreased number of fetuses and increased abnormal scarring compared to CB-pregnant, suggesting GDM-associated miscarriage. This result might be associated with the higher BGL at GD7.5, because GD7.5 is the placentation phase immediately after implantation at GD6.5. The author also explored the relationship between high BGL tendency from GD7.5 and the decreased number of fetuses due to imbalanced glucose distribution in STZ-pregnant. However, hyperglycemia in the STZ-pregnant group did not increase fetal weight, unlike the macrosomia found in human GDM. One reason for these species-specific differences may be the effects of multiple births in mice, which could result in less glucose distribution in later pregnancy stages, similar to observations in human twin pregnancies [34]. In contrast, although mice and humans show hemochorial placental features, the layer structures of trophoblastic cells expressing GLUTs differ among species, with three layers in mice and single in humans [35, 36]. Therefore, these species-specific differences in placental structures may contribute to variations in glucose transport to the fetus, both in pregnancies and in pregnancies affected by GDM.

The kidney in DM shows glomerular, tubulointerstitial, and vascular lesions due to hyperglycemia, and pregnancy also increases the risk of kidney disease and exacerbates its pathology by increasing the GFR in humans [37]. Changes related to a higher BGL were observed in the kidneys of STZ-virgin mice, and elevated expression of *Il6* and *Vegfa* was noted. Proximal tubular epithelial cells damaged by high glucose secrete vascular endothelial growth factor A (VEGFA) to alleviate this disorder in mice [38]. For IL-6, its serum level increases in human patients with type 2 diabetes [39], and obesity-based GDM mice also show renal damage, with increased blood IL-6 levels and activated MAPK signaling in the kidney [15]. In contrast, severe histopathological changes were not observed in the STZ-virgin or STZ-pregnant groups, except for vacuoles or deciduation of renal tubular epithelial cells, and the renal expression of *Il6* and *Vegfa* tended to be lower in the STZ-pregnant group than in the STZ-virgin group. Therefore, the intraperitoneal STZ administration method used in this study did not lead to significant renal damage.

The renal threshold for glucose is influenced by factors such as renal blood flow and the expression of GLUT2 or SGLT2 in the proximal tubule [11]. It typically increases in DM and decreases during pregnancy. The kidney weight increased in the CB-pregnant group, but not in the STZ-pregnant group, indicating that the kidney structure in STZ-pregnant might not undergo changes due to increased renal blood flow associated with fetuses. In fact, the expression of GLUT2 and SGLT2 (encoded by *Slc2a2* and *Slc5a2*, respectively) did not differ between CB-pregnant and STZ-pregnant. Considering urinary glucose was observed similarly in both groups, the balance between BGL increase and glucose excretion might be comparable between CB-pregnant and STZ-pregnant. Given that the GDM phenotype would also be milder in STZ-pregnant compared to representative GDM patients or obesity-based GDM models [40], the GDM kidney in non-obese status might have a reserve capacity to adapt to changes in the renal threshold for glucose during DM and pregnancy.

In a previous human study, lipid metabolism was observed to undergo changes to facilitate fat storage in the early stages of pregnancy, characterized by decreases in triacylglycerols and total or LDL cholesterol, along with an increase in HDL cholesterol levels in the blood [41]. Conversely, late pregnancy is associated with changes in lipid metabolism to promote fat mobilization, leading to increases in triacylglycerols and total LDL or HDL cholesterol levels [41]. These alterations may be attributed to the elevated estrogen concentration and insulin resistance typically observed during pregnancy. However, GDM is characterized by dyslipidemia, presenting with elevated triacylglycerol levels and reduced LDL cholesterol concentrations in the blood [42]. Mice with GDM induced by high-fat and high-sugar diets have been shown to exhibit hepatic lipid droplet accumulation due to a reduction in triacylglycerol and LDL levels, accompanied by increased insulin resistance [16]. Additionally, GDM mice deficient in adiponectin exhibit higher triacylglycerol levels and increased lipid droplets in the liver [43]. In our study, pregnant mice demonstrated a significant increase in ADRP/Perilipin 2+ lipid droplets in the liver, regardless of STZ i.p. administration, indicating that these changes occurred during pregnancy rather than because of diabetic status.

The gene expression of insulin function-related molecules in the liver was significantly lower in the STZ-pregnant group than in the CB-pregnant group, which contributed to enhanced insulin resistance. In contrast, the liver histology and glucose transporter expression were similar in the CB-pregnant and STZ-pregnant groups. As for the gene expression pattern differences between CB-pregnant and STZ-pregnant, IGF2R (encoded by *Igf2r*) was increased in the duodenum of the STZ-pregnant group. IGF-1 and IGF-2 increase insulin sensitivity and improve glucose metabolism via IGF-1R, whereas IGF2R degrades IGF2 to inhibit its function [44]. IGF2 polymorphism is associated with GDM risk in humans [45]. Therefore, altered *Igf2r* expression in duodenum might reflect or be related to insulin resistance in patients with GDM. Furthermore, the expression of GLUT5 (*Slc2a5*) and GLUT12 (*Slc2a12*) was decreased in the duodenum of STZ-pregnant mice. GLUT5 is crucial for fructose transport in the intestine, and GLUT12 in humans is an insulin-sensitive transporter of glucose related to fat and uric acid [46]. Therefore, not only the main organs related to glucose metabolism, such as the kidney and liver, but also other organs, such as the intestines, contribute to the clinicopathological or adaptive changes in GDM through the alteration of the insulin/IGF axis and/or glucose transportation.

In conclusion, this study contributes to the establishment of a GDM mouse model that closely mimics human GDM pathology, thereby aiding in the elucidation of GDM pathogenesis. Furthermore, it suggests that a predisposition to glucose intolerance in virgin mice may serve as a risk factor for GDM. These finding sheds light on non-obese patients with GDM, a population that has not been extensively studied previously, and highlights the potential relevance of a predisposition to glucose intolerance in the virgin state, similar to what was observed in this mouse model. Considering that mice also develop GDM, which can have adverse effects on both the mother and fetus, it is conceivable that GDM cases might exist in several animal species. Moreover, this study underscores the importance of monitoring and regulating metabolic indicators, including blood glucose levels and cholesterol, to manage unexplained reproductive disorders in both humans and animals. Additionally, the author emphasizes the significance of considering species-specific differences in anatomy and physiology. Even if the pathogenesis of GDM is similar in humans and animals, the effects on terminal organs may vary among species. Therefore, a thorough understanding of these species-specific differences is essential for accurately interpreting and translating research findings from animal models to clinical settings.

## Supporting information

S1 Table Correlation of BGLs with reproductive ability. (DOCX)

S1 Fig Genes associated with insulin sensitivity and glucose transportation. (A) mRNA expression of genes of insulin receptor. (B) mRNA expression of genes of glucose transporter. Significant differences: CV: *P* < 0.05, citrate buffer (CB) injected virgin vs streptozotocin (STZ) injected virgin. CV: *P* < 0.05, CB virgin vs CB pregnant. SV: *P* < 0.05, STZ virgin vs STZ pregnant. CP: *P* < 0.05, CB pregnant vs STZ pregnant, Mann-Whitney *U*-test.
(PDF)
