# Supplementary material for: Phenotypes of streptozotocin-induced gestational diabetes mellitus in mice
Source: PLoS One. 2024 Apr 16;19(4):e0302041. doi: 10.1371/journal.pone.0302041 (PMC11020761; doi:10.1371/journal.pone.0302041)
Supplement: S1 Table — (DOCX) [file pone.0302041.s001.docx]

**Supplemental table 1. Correlation of BGLs with reproductive ability.**

| **Time point (GD)** | **Group** | **No. fetuses** | | **No. absorption scars** | | **Fetus weight** | | **No. corpora lutea** | |
| --- | --- | --- | --- | --- | --- | --- | --- | --- | --- |
|  |  | *ρ* | *P* | *ρ* | *P* | *ρ* | *P* | *ρ* | *P* |
| 1.5 | CB | −0.949 | 0.051 | NA | NA | −1.000 | NA | 1.000** | NA |
|  | STZ | 0.000 | 1.000 | −0.205 | 0.741 | 0.700 | 0.188 | −0.100 | 0.873 |
|  | All | −0.077 | 0.845 | −0.064 | 0.891 | 0.505 | 0.248 | 0.217 | 0.606 |
| 3.5 | CB | −0.738 | 0.262 | NA | NA | −1.000 | NA | 1.000** | NA |
|  | STZ | 0.000 | 1.000 | −0.410 | 0.493 | 0.800 | 0.104 | 0.100 | 0.873 |
|  | All | −0.152 | 0.696 | 0.055 | 0.908 | 0.357 | 0.432 | 0.419 | 0.301 |
| 5.5 | CB | −0.211 | 0.789 | NA | NA | 1.000 | NA | −0.500 | 0.667 |
|  | STZ | 0.000 | 1.000 | −0.410 | 0.493 | 0.800 | 0.104 | 0.100 | 0.873 |
|  | All | 0.267 | 0.487 | −0.455 | 0.305 | 0.643 | 0.119 | 0.181 | 0.668 |
| 7.5 | CB | 0.316 | 0.684 | NA | NA | 1.000 | NA | −1.000** | NA |
|  | STZ | −0.369 | 0.541 | -0.154 | 0.805 | 0.300 | 0.624 | −0.100 | 0.873 |
|  | All | −0.430 | 0.248 | 0.382 | 0.398 | 0.571 | 0.18 | −0.048 | 0.910 |
| 9.5 | CB | −0.211 | 0.789 | NA | NA | −1.000 | NA | 0.500 | 0.667 |
|  | STZ | −0.158 | 0.800 | -0.103 | 0.870 | 0.500 | 0.391 | −0.300 | 0.624 |
|  | all | −0.177 | 0.648 | 0.327 | 0.474 | 0.286 | 0.535 | −0.120 | 0.778 |
| 11.5 | CB | 0.632 | 0.368 | NA | NA | −1.000 | NA | −0.500 | 0.667 |
|  | STZ | −0.264 | 0.668 | 0.616 | 0.269 | −0.600 | 0.285 | −0.200 | 0.747 |
|  | all | −0.025 | 0.948 | 0.218 | 0.638 | −0.679 | 0.094 | −0.240 | 0.568 |
| 13.5 | CB | −0.211 | 0.789 | NA | NA | −1.000 | NA | 0.500 | 0.667 |
|  | STZ | −0.580 | 0.306 | 0.821 | 0.089 | −1.000** | NA | −0.600 | 0.285 |
|  | All | −0.608 | 0.083 | .855* | 0.014 | −0.500 | 0.253 | −0.347 | 0.399 |
| 15.5 | CB | −0.738 | 0.262 | NA | NA | 1.000 | NA | 0.500 | 0.667 |
|  | STZ | −0.738 | 0.155 | 0.718 | 0.172 | −0.500 | 0.391 | −.900* | 0.037 |
|  | All | −-.836** | 0.005 | .891** | 0.007 | 0.107 | 0.819 | −0.347 | 0.399 |
| 17.5 | CB | 0.632 | 0.368 | NA | NA | 1.000 | NA | −1.000** | NA |
|  | STZ | 0.000 | 1.000 | −0.205 | 0.741 | 0.700 | 0.188 | −0.100 | 0.873 |
|  | All | −0.405 | 0.279 | 0.418 | 0.350 | 0.607 | 0.148 | −0.335 | 0.417 |
| Spearman’s lank correlation coefficients. *: *P* < 0.05, **: *P* < 0.01. n > 4 (CB), n=5 (STZ). BGL, blood glucose level; GD, gestational days; No., the number of; NA, not applicable. | | | | | | | | | |
