# Supplementary material for: Phenotypes of streptozotocin-induced gestational diabetes mellitus in mice
Source: PLoS One. 2024 Apr 16;19(4):e0302041. doi: 10.1371/journal.pone.0302041 (PMC11020761; doi:10.1371/journal.pone.0302041)
Supplement: S1 Fig — (A) mRNA expression of genes of insulin receptor. (B) mRNA expression of genes of glucose transporter. Significant differences: CV: P < 0.05, citrate buffer (CB) injected virgin vs streptozotocin (STZ) injected virgin. CV: P < 0.05, CB virgin vs CB pregnant. SV: P < 0.05, STZ virgin vs STZ pregnant. CP: P < 0.05, CB pregnant vs STZ pregnant, Mann-Whitney U-test. (PDF) [file pone.0302041.s002.pdf]

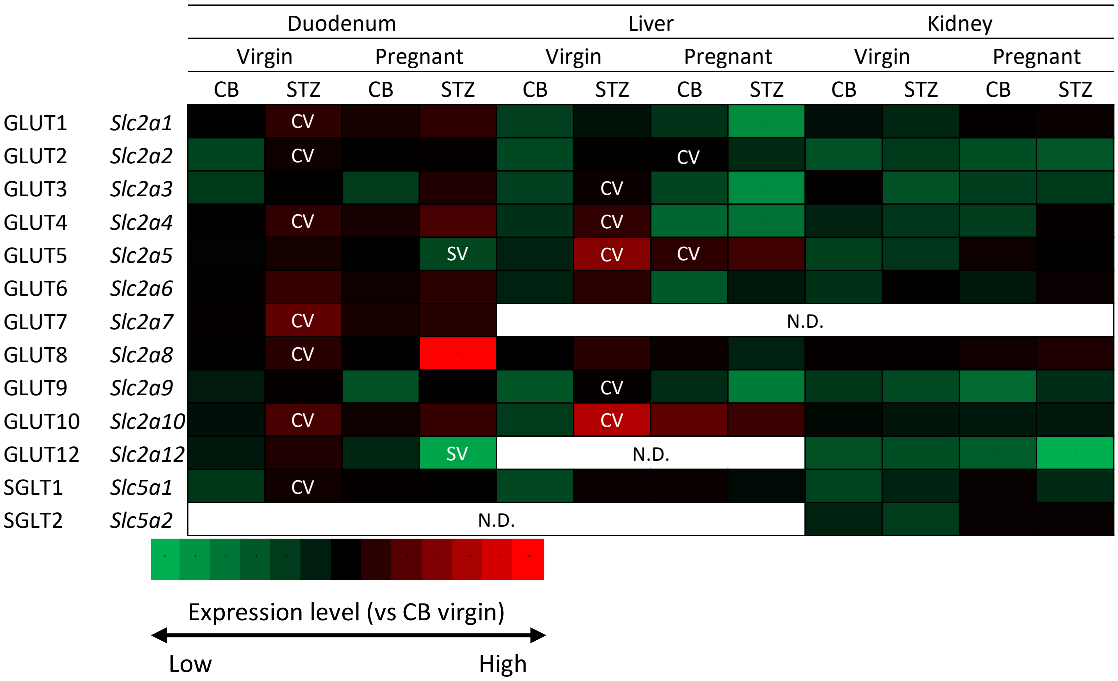


**Supplemental figure 1. Genes associated with glucose transportation.**
Significant differences: CV: *P* < 0.05, citrate buffer (CB) injected virgin vs streptozotocin (STZ) injected virgin. CV: *P* < 0.05, CB virgin vs CB pregnant. SV: *P* < 0.05, STZ virgin vs STZ pregnant. CP: *P* < 0.05, CB pregnant vs STZ pregnant, Mann-Whitney *U*-test.
